# Prediction equations for digestible and metabolizable energy concentrations in feed ingredients and diets for pigs based on chemical composition

**DOI:** 10.5713/ajas.20.0293

**Published:** 2020-07-14

**Authors:** Jung Yeol Sung, Beob Gyun Kim

**Affiliations:** 1 Department of Animal Science and Technology, Konkuk University, Seoul 05029, Korea

**Keywords:** Digestible Energy, Metabolizable Energy, Prediction Equation, Swine

## Abstract

**Objective:**

The objectives were to develop prediction equations for digestible energy (DE) and metabolizable energy (ME) of feed ingredients and diets for pigs based on chemical composition and to evaluate the accuracy of the equations using *in vivo* data.

**Methods:**

A total of 734 data points from 81 experiments were employed to develop prediction equations for DE and ME in feed ingredients and diets. The CORR procedure of SAS was used to determine correlation coefficients between chemical components and energy concentrations and the REG procedure was used to generate prediction equations. Developed equations were tested for the accuracy according to the regression analysis using *in vivo* data.

**Results:**

The DE and ME in feed ingredients and diets were most negatively correlated with acid detergent fiber or neutral detergent fiber (NDF; r = −0.46 to r = −0.67; p<0.05). Three prediction equations for feed ingredients reflected *in vivo* data well as follows: DE = 728+ 0.76×gross energy (GE)−25.18×NDF (R^2^ = 0.64); ME = 965+0.66×GE−24.62×NDF (R^2^ = 0.60); ME = 1,133+0.65×GE−29.05×ash−23.17×NDF (R^2^ = 0.67).

**Conclusion:**

In conclusion, the equations suggested in the current study would predict energy concentration in feed ingredients and diets.

## INTRODUCTION

Energy is known to be one of the most expensive nutritional components in animal feeds. Available energy of feeds can be partitioned into digestible energy (DE), metabolizable energy (ME), and net energy subtracting energy losses from gross energy (GE) [1]. However, net energy is less widely used compared with DE and ME due to difficulties of its determination and relatively large variations [2]. To obtain energy concentrations of feed ingredients and diets, metabolism experiments are required to collect feces and urine through restriction of physical activity of animals. However, as *in vivo* experiments are time–consuming and costly, prediction equations based on chemical components have been regarded as an alternative method for obtaining DE and ME of feeds [3].

Prediction equations for DE or ME for pigs have been developed for specific feed ingredients [4–6] and diets [3,7,8]. However, available equations for predicting energy concentrations in the literature are not applicable or risky in many cases. For example, some equations can be applied only to specific ingredients and others have a relatively narrow range of dietary chemical compositions. Inappropriate uses of these equations are susceptible to extrapolation bias [9,10]. To solve these problems, Choi et al [11] developed equations for DE in feed ingredients and diets for growing pigs and these equations were shown to be more accurate than previously published equations. However, Choi et al [11] provided equations only for DE but not ME. Therefore, the objectives of this study were to develop widely applicable prediction equations for DE and ME in feed ingredients and diets for pigs based on chemical components and to validate these equations using data from *in vivo* experiments.

## MATERIALS AND METHODS

### Data collection

A total of 734 data points from 81 studies that determined DE or ME were employed to develop equations for energy concentrations in feed ingredients and diets for pigs. The database consisted of chemical compositions and energy concentrations of feed ingredients and diets used in the experiments. Energy contents were expressed in kcal/kg dry matter (DM) and other variables were in % DM. When chemical components of feed ingredients or diets were not provided in a paper, chemical components were calculated by multiplying the inclusion rate of each ingredient by values provided in Sauvant et al [12] and NRC [1].

### Statistical analysis

Correlation coefficients between chemical components and energy concentrations were determined by the CORR procedure of SAS (SAS Inst. Inc., Cary, NC, USA). Prediction equations for DE and ME were developed by the REG procedure. A coefficient of determination was an indicator to define the best fit equations.

The alpha level used for determining statistical significance was 0.05. The accuracy of prediction equations for energy concentrations was assessed by regressing the determined values of data from *in vivo* experiments [13–18] minus the predicted values for energy concentrations on the predicted values centered to the mean [19]. These data had not been used in developing prediction equations. To validate prediction equations for energy concentrations, only data corresponding to the range of chemical components used for equations in the current study were employed.

## RESULTS AND DISCUSSION

Both of index method and total collection method are used for determining DE in feed ingredients and diets in pigs. In the present work, the experiments that used the index method were excluded. The reason for excluding data from the index method was that the indigestible index method may potentially underestimate nutrient digestibility due to low index recovery [20].

Feed ingredients had a wider range and greater variations of chemical components compared with diets (Table 1). The range of chemical composition of feed ingredients in the current study was wider than that of the previous studies [6,8,11], which is desirable for establishing prediction equations. The range of dietary chemical components of diets in the present work was wider than that in the work by Noblet and Perez [3], but maximum ash, crude protein, and neutral detergent fiber (NDF) contents were less than those of previous studies [7,8].

The DE and ME in feed ingredients and diets were correlated most negatively with acid detergent fiber (ADF) and NDF representing dietary fiber (r = −0.46 to r = −0.67; Tables 2, 3). The negative correlations between dietary fibers and available energy are in good agreement with previous studies where dietary fiber was the best predictor [3,7,8,11]. This is because dietary fiber decreases energy digestibility of other nutrients, and the digestibility of fiber itself is relatively less compared with starch, proteins, and lipids [7]. Dietary fiber can be categorized into crude fiber, ADF, NDF, total dietary fiber (TDF), insoluble dietary fiber, and soluble dietary fiber based on fiber analysis procedures [1]. In some previous studies, TDF was the most accurate independent variable to predict DE in corn byproducts among various dietary fibers [21,22]. As TDF includes β-glucans unlike other dietary fibers, theoretically, TDF provides an accurate estimate of dietary fiber [1]. However, a recent meta-analysis study indicated that dietary fiber analyzed by detergent fiber procedure showed a greater accuracy in predicting DE in feed ingredients and diets compared with TDF, insoluble dietary fiber, and soluble dietary fiber [11]. For this reason, the NDF and ADF were used to represent dietary fiber when developing equations in the current study.

Ash was also regarded as a potential independent variable for estimating DE and ME in feed ingredients and diets in the present work. Generally, energy concentrations in ingredients and diets decrease as ash concentration increases due to the lack of GE in ash [3]. In the current study, ash was negatively correlated with GE (r = −0.25), DE (r = −0.33), and ME (r = −0.45) in feed ingredients and was used for predicting DE and ME in several equations (Eq. 3, 5, 7, 8, and 12 to 17). In contrast to feed ingredients, ash was not included in the equations for predicting DE and ME in diets, which is likely due to the relatively narrow range of ash contents in diets (2.3% to 11.7%) compared with feed ingredients (0.2% to 46.2%).

Based on coefficients of determination, prediction equations for DE and ME in feed ingredients and diets were developed (Tables 4, 5). Equations with less than 0.5 of coefficient of determination were excluded. Some variables were highly correlated each other resulting in a decrease of the validity of regression coefficients as predictors [7]. For this reason, ADF and NDF were not included in the equation as independent variables as well as ether extract and GE.

*In vivo* experiments for testing the accuracy of the present equations were conducted in our laboratory and experimental conditions were kept constant among experiments (e.g., chemical analysis, environment and facilities, experimental procedures, and genetic strains of pigs). The DE or ME obtained from *in vivo* experiments was plotted against the DE or ME calculated using the equations developed in the present work. The intercept and slope generated by mean-centered regression representing a mean bias and linear bias, respectively, can be used as indicators of accuracy [23]. The mean bias and linear bias represent the difference between the average of measured and predicted energy values and the consistency of prediction error across the range of data, respectively. Based on the regression analyses, the slope and intercept were not different from 0 when validating Eq. 2, 11, and 12 (Tables 4, 5), which indicates that these equations accurately estimates *in vivo* DE and ME data employed in the present work.

Compared with prediction models for DE or ME in the literature, the present equations showed lower coefficients of determination and greater root mean squares of error. One possible reason is that experiments used to establish equations in the current study were conducted by various experimental stations. Experimental conditions may differ among institutions and these factors may cause variations. Particularly for chemical analysis, statistically significant difference may occur among analyzed values even though each station follows the same methods of chemical analysis [24]. In contrast to the current study, factors aforementioned were relatively controlled in previous modeling studies because most of metabolism experiments and chemical analysis were conducted in their own laboratories [3,6,25]. Another possible reason is that most of data used for establishing equations in the current study were derived from energy evaluation of feed ingredients. To evaluate available energy concentration in a feed ingredient precisely, the proportion of a target feedstuff is high which leads to an impractical diet formulation. In contrast, most diets fed to pigs to derive equations in previous studies [3,26] were corn- or wheat-based diets where the proportion of non-conventional feed ingredients was low. In addition, while previous equations for feed ingredients were applicable for specific feed ingredients such as animal byproduct, corn coproduct, and wheat [6,21,27], the equations developed in the present work were established based on various types of feed ingredients.

Despite these problems, the equations suggested in the current study are meaningful because most of data used for developing equations were derived from the recent studies. A great number of recent observations employed in the present work compared with previous studies should also be noted [3,7,25]. Furthermore, independent variables in equations consisted of commonly analyzed components in laboratories and equations were based on various combinations of chemical components.

## CONCLUSION

Based on chemical compositions, energy concentrations in feed ingredients and diets can be fairly accurately estimated using the prediction equations proposed in this study. Further studies are warranted to develop prediction equations for net energy.

## Figures and Tables

**Table 1:** The variability of chemical components in feed ingredients and diets in the database (dry matter basis)

Item	Feed ingredients					Diets				
	
	n	Mean	Min.	Max.	SD	n	Mean	Min.	Max.	SD
GE (kcal/kg)	423	4,794	3,317	6,349	491	278	4,489	4,025	5,319	211
DE (kcal/kg)	438	3,733	1,856	6,099	631	283	3,810	3,007	4,501	289
ME (kcal/kg)	437	3,497	1,569	5,989	606	264	3,655	2,893	4,321	279
EE (%)	397	5.8	0	27.5	4.9	215	4.2	0.6	11.8	2.2
CP (%)	450	32.9	2.0	101.4	20.8	282	18.6	7.3	30.1	5.2
NDF (%)	402	25.8	0	84.8	14.5	269	14.9	2.6	51.7	6.8
ADF (%)	395	10.6	0	53.8	8.4	239	5.8	1.6	22.2	3.1
Ash (%)	416	7.0	0.2	46.2	7.1	177	5.3	2.3	11.7	1.5

SD, standard deviation; GE, gross energy; DE, digestible energy; ME, metabolizable energy; EE, ether extract; CP, crude protein; NDF, neutral detergent fiber; ADF, acid detergent fiber.

**Table 2:** Correlation coefficients between energy concentrations and chemical components in feed ingredients

Item	GE	DE	ME	EE	CP	NDF	ADF
DE	0.52***	-	-	-	-	-	-
ME	0.48***	0.97***	-	-	-	-	-
EE	0.45***	0.08	0.00	-	-	-	-
CP	0.37***	0.31***	0.14**	0.05	-	-	-
NDF	0.08***	−0.57***	−0.58***	0.33***	−0.17***	-	-
ADF	0.12*	−0.58***	−0.54***	0.12*	0.03	0.77***	-
Ash	−0.25***	−0.33***	−0.45***	0.40***	0.36***	0.15**	0.12*

GE, gross energy; DE, digestible energy; ME, metabolizable energy; EE, ether extract; CP, crude protein; NDF, neutral detergent fiber; ADF, acid detergent fiber.

* p<0.05,

** p<0.01, and

*** p<0.001.

**Table 3:** Correlation coefficients between energy concentrations and chemical components in diets

Item	GE	DE	ME	EE	CP	NDF	ADF
DE	0.37***	-	-	-	-	-	-
ME	0.20***	0.98***	-	-	-	-	-
EE	0.61***	0.12	0.05	-	-	-	-
CP	0.29***	−0.01	−0.11	0.03	-	-	-
NDF	0.19**	−0.46***	−0.52***	0.30***	0.09	-	-
ADF	0.16*	−0.63***	−0.67***	0.27***	0.12	0.77***	-
Ash	0.27***	−0.12	−0.31***	0.24**	0.28***	0.22**	0.30***

GE, gross energy; DE, digestible energy; ME, metabolizable energy; EE, ether extract; CP, crude protein; NDF, neutral detergent fiber; ADF, acid detergent fiber.

* p<0.05,

** p<0.01, and

*** p<0.001.

**Table 4:** Prediction equations for energy concentrations in feed ingredients and validation of equations1),2)

Equation No.	n	Equation3)	RMSE	R^2^	Intercept4)		Slope5)	
	
					Value	p-value	Value	p-value
1	364	DE = 398+0.79×GE−44.90×ADF	355	0.66	27 (70)	0.701	−0.29 (0.13)	0.028
2	371	DE = 728+0.76×GE−25.18×NDF	363	0.64	−49 (63)	0.442	−0.22 (0.12)	0.066
3	343	DE = 536+0.84×GE−42.33×ash−23.87×NDF	330	0.71	−33 (62)	0.590	−0.25 (0.11)	0.025
4	344	DE = 3,558+14.49×CP+52.06×EE−54.68×ADF	354	0.66	43 (72)	0.555	−0.44 (0.11)	<0.001
5	353	DE = 3,901−40.07×ash+14.71×CP−40.79×ADF	411	0.55	−5 (75)	0.952	−0.40 (0.13)	0.004
6	351	DE = 3,882+8.78×CP+55.38×EE−29.05×NDF	397	0.56	−56 (65)	0.391	−0.30 (0.11)	0.008
7	320	DE = 3,665−57.10×ash+17.86×CP+50.57×EE−47.72×ADF	328	0.71	84 (68)	0.225	−0.40 (0.10)	0.001
8	329	DE = 3,969−80.49×ash+14.37×CP+66.42×EE−25.19×NDF	325	0.71	−1 (59)	0.983	−0.30 (0.09)	0.002
9	425	ME = 0.97×DE−3.86×CP	120	1.00	43 (10)	<0.001	0.06 (0.02)	0.001
10	369	ME = 568+0.71×GE−43.30×ADF	379	0.58	73 (78)	0.355	−0.32 (0.15)	0.038
11	372	ME = 965+0.66×GE−24.62×NDF	370	0.60	−23 (73)	0.754	−0.26 (0.14)	0.072
12	345	ME = 1,133+0.65×GE−29.05×ash−23.17×NDF	338	0.67	−26 (69)	0.713	−0.21 (0.13)	0.112
13	342	ME = 781+0.69×GE−30.88×ash−40.87×ADF	347	0.66	66 (75)	0.384	−0.29 (0.14)	0.044
14	331	ME = 4,178−48.19×ash+60.43×EE−28.79×NDF	355	0.63	−23 (64)	0.721	−0.26 (0.11)	0.018
15	361	ME = 3,854−56.40×ash+11.12×CP−38.36×ADF	399	0.53	36 (80)	0.655	−0.41 (0.14)	0.007
16	328	ME = 3,662−72.14×ash+13.73×CP+46.32×EE−46.24×ADF	327	0.69	120 (75)	0.117	−0.41 (0.12)	0.001
17	331	ME = 3,903−64.52×ash+8.70×CP+64.49×EE−26.43×NDF	321	0.70	9 (66)	0.897	−0.28 (0.11)	0.014

RMSE, root mean square of error; DE, digestible energy; GE, gross energy; ADF, acid detergent fiber; NDF, neutral detergent fiber; CP, crude protein; EE, ether extract; ME, metabolizable energy.

1) Thirty nine *in vivo* data points were employed to validate prediction equations for energy concentrations in feed ingredients.

2) Values in parentheses are standard error.

3) Energy concentrations and chemical composition are expressed as kcal/kg dry matter and % dry matter, respectively.

4) The intercept represents the mean bias.

5) The slope represents the linear bias.

**Table 5:** Prediction equations for energy concentrations in diets and validation of equations1),2)

Equation No.	n	Equation3)	RMSE	R^2^	Intercept4)		Slope5)	
	
					Value	p-value	Value	p-value
18	232	DE = 965+0.72×GE−66.40×ADF	187	0.61	−13 (28)	0.646	−0.70 (0.11)	<0.001
19	262	ME = 0.98×DE−4.22×CP	55	1.00	31 (4)	<0.001	0.06 (0.02)	0.011
20	228	ME = 1,521+0.56×GE−65.30×ADF	184	0.59	21 (29)	0.470	−0.71 (0.11)	<0.001
21	185	ME = 3,847+38.69×EE−67.24×ADF	181	0.57	41 (30)	0.174	−0.77 (0.11)	<0.001
22	185	ME = 3,741+5.75×CP+39.84×EE−68.86×ADF	179	0.58	63 (29)	0.041	−0.76 (0.10)	<0.001

RMSE, root mean square of error; DE, digestible energy; GE, gross energy; ADF, acid detergent fiber; ME, metabolizable energy; CP, crude protein; EE, ether extract.

1) Thirty two *in vivo* data points were employed to validate prediction equations for energy concentrations in feed ingredients.

2) Values in parentheses are standard error.

3) Energy concentrations and chemical composition are expressed as kcal/kg dry matter and % dry matter, respectively.

4) The intercept represents the mean bias.

5) The slope represents the linear bias.
